# Ginkgolic Acid Rescues Lens Epithelial Cells from Injury Caused by Redox Regulated-Aberrant Sumoylation Signaling by Reviving Prdx6 and Sp1 Expression and Activities

**DOI:** 10.3390/ijms19113520

**Published:** 2018-11-08

**Authors:** Bhavana Chhunchha, Prerna Singh, Dhirendra P. Singh, Eri Kubo

**Affiliations:** 1 Department of Ophthalmology and Visual Science, University of Nebraska Medical Center, Omaha, NE 68198, USA; prernasingh10@gmail.com (P.S.); dpsingh@unmc.edu (D.P.S.); 2 Department of Ophthalmology, Kanazawa Medical University, Ishikawa 9200293, Japan

**Keywords:** oxidative stress, Sumo1, ginkgolic acid, betulinic acid, Prdx6, Sp1

## Abstract

Sumoylation is a downstream effector of aging/oxidative stress; excess oxidative stress leads to dysregulation of a specificity protein1 (Sp1) and its target genes, such as Peroxiredoxin 6 (Prdx6), resulting in cellular damage. To cope with oxidative stress, cells rely on a signaling pathway involving redox-sensitive genes. Herein, we examined the therapeutic efficacy of the small molecule Ginkgolic acid (GA), a Sumoylation antagonist, to disrupt aberrant Sumoylation signaling in human and mouse lens epithelial cells (LECs) facing oxidative stress or aberrantly expressing Sumo1 (small ubiquitin-like modifier). We found that GA globally reduced aberrant Sumoylation of proteins. In contrast, Betulinic acid (BA), a Sumoylation agonist, augmented the process. GA increased Sp1 and Prdx6 expression by disrupting the Sumoylation signaling, while BA repressed the expression of both molecules. In vitro DNA binding, transactivation, Sumoylation and expression assays revealed that GA enhanced Sp1 binding to GC-boxes in the Prdx6 promoter and upregulated its transcription. Cell viability and intracellular redox status assays showed that LECs pretreated with GA gained resistance against oxidative stress-driven aberrant Sumoylation signaling. Overall, our study revealed an unprecedented role for GA in LECs and provided new mechanistic insights into the use of GA in rescuing LECs from aging/oxidative stress-evoked dysregulation of Sp1/Prdx6 protective molecules.

## 1. Introduction

The posttranslational modification Sumoylation of proteins to a specific lysine (K) residue in target protein by the small ubiquitin-related modifier (SUMO) protein family regulates diverse cellular processes, including protein stabilization, gene transcription, regulation of protein function, protein-protein and protein-DNA interaction and so forth. In doing so, Sumoylation maintains cellular homeostasis [1,2,3]. Three major Sumo isoforms, Sumo1, Sumo2 and Sumo3, have been reported in mammals [4,5]; Sumo4 and Sumo5 have been found to be organ-specific [6,7,8]. A three-enzyme cascade is involved in the Sumoylation process: the E1-activating complex SAE1/SAE2, E2-conjugating enzyme UBC9 and E3 ligases, in an ATP-dependent manner. For in vitro Sumoylation, activating enzyme E1 and conjugating enzyme E2 are sufficient, whereas both in vivo and in vitro Sumo conjugation are facilitated by Sumo ligase E3s [1,9,10]. Coordinated activity of these enzymes supports the interaction of Sumo with Sumo-interacting site(s) present in target proteins [11], and an imbalance can lead to erratic Sumoylation. The Sumoylation process can be disturbed by various physiological and environmental stressors. Among these are oxidative stress caused by overproduction of intracellular reactive oxygen species (ROS) in response to external stimuli and loss of antioxidant proteins in aging [12,13,14,15]. Furthermore, emerging evidence indicates that excess oxidative stress leads to aberrant Sumoylation, which results in abnormal functions of nuclear or extra-nuclear proteins. Cellular Sumoylation level also controls cellular fate by modulating protein stability, which affects the availability of intracellular proteins, such as transcription factors and their target proteins responsible for cellular protection [13,14,16,17,18,19,20,21]. Recent evidence reveals that a disturbance in the Sumoylation process results in the onset and progression of several disorders [22,23,24,25,26]. Based on these facts, we hypothesized that blocking aberrant Sumoylation by means of Sumoylation inhibitors should be a logical strategy to reverse aberrant Sumoylation-mediated pathobiology of cells/tissues by restoring the cellular proteins responsible for maintaining cell homeostasis.

Oxidative stress caused by an excess of ROS production can affect the highly dynamic process of Sumoylation by altering the activity of a Sumo-specific protease (Senps) responsible for the deSumoylation process [9,27]. All seven members of the Senp family share 20–60% sequence identity within their catalytic domain [28]. Senp1 is mostly responsible for the deconjugation of both Sumo1 and Sumo2/3 modifications in a large number of target proteins, and thus, is involved in many cellular processes. Our group and others have reported that Senp1 regulates the activity of many transcription factors, including specificity protein1 (Sp1) in vivo and in vitro [9,13,14,17,29]. The Senp family regulates Sumo homeostasis in eukaryotic cells [30]. Dysregulation or imbalance between the Sumoylation and deSumoylation processes has been implicated in a wide variety of degenerative diseases [4]. During aging and oxidative stress, ROS are continuously accumulated with a decline in expression of antioxidant proteins, such as Peroxiredoxin 6 (Prdx6), super oxide dismutase, catalase, etc. [14,15]. Notably, Senps are known to be redox sensitive and effectors, and their activity can be affected by oxidative stress. Levels of ROS can modify Senp1 and promote a reversible Senp1 blockade by formation of an intermolecular disulfide bond through the active site Cysteine 603 and a unique residue Cysteine 613. Overproduction and accumulation of intracellular ROS can irreversibly inactivate Senps due to their dimerization, and that ultimately may lead to aberrant Sumoylation of proteins [13,14,31], to dysregulation of such proteins as Sp1, IkB, Prdx6 and so on, and to failure of cellular antioxidant homeostasis and cell death.

A newly discovered family of antioxidant enzymes, the Peroxiredoxins (Prdxs), is widely distributed [32,33]. The Prdx family is divided into two categories, 1-Cys and 2-Cys, based on the number of cysteine (Cys) residues. Prdxs 1–5 are 2-Cys residues, while Prdx6 is 1-Cys Prdx, which lacks a COOH-terminal Cys residue. Prdx6 is a unique member of the Prdx family, as it contains glutathione (GSH) peroxidase and calcium independent phospholipase A_2_ (PLA_2_) activities, and multifunctional proteins. [34,35,36,37]. Prdx6 is widely expressed throughout all organs with especially high concentrations in the eye lens, lung, brain, liver, kidney and testis [12,34,38], wherein Prdx6 maintains cellular homeostasis by maintaining ROS levels. Loss of Prdx6 in cells or tissues has been shown to cause cell injuries related to oxidative stress. Thus, Prdx6 expression is vitally important to cellular survival. 

Recently, we reported that Prdx6 is transcriptionally regulated by transcription factor Sp1, a member of the zinc finger family of transcription factors [12,13,14]. Sp1 is a stress-inducible, anti-death transcription factor with a wide variety of beneficial activities, which it exerts by binding to GC-rich elements (or GC box). Sp1 is involved in many cellular processes, including cell differentiation, cell growth, apoptosis, immune responses, response to DNA damage and chromatin remodeling. Post-translational modifications, such as Sumoylation, phosphorylation, glycosylation, acetylation, etc. and proteolytic processing significantly affect the activity of Sp1, which can act as an activator or a repressor. Additionally, Sp1 is auto-regulated. We previously reported that aging lens/lens epithelial cells (LECs) and LECs facing oxidative stress showed significant reduction in Sp1 activity and expression [12,13,14]. Sumoylation of Sp1 has been shown to be involved in its dysregulation. We posit that aberrant Sumoylation-mediated Sp1 dysregulation can repress its own expression as well as expression of its target genes, such as Prdx6. 

During aging and oxidative stress, Sumo1 conjugation increases [13,20,39] due to aberrant Sumoylation signaling in redox-active cells. This aberrant process is thought to be a major culprit in the pathobiology of tissues. Very recently, we have shown that Sumoylation-deficient Sp1 K (lysine) 16 R (arginine) conferred resistance against oxidative stress-induced hyperSumoylation, and its ectopic expression promoted cell survival during oxidative stress and aberrant Sumoylation-mediated death signaling by upregulating protective protein Prdx6 [40]. However, recently, efforts have been made to develop small deliverable (and druggable) molecules, such as Ginkgolic acid (GA) [4], that have the ability to inhibit the aberrant Sumoylation process and postpone pathobiology. GA is a prominent botanical drug extracted from the leaves and seed coat of *Ginkgo biloba* L. GA has a wide range of bioactive properties with beneficial effects on neurological and cardiovascular diseases, cancer, age-related macular degeneration, Alzheimer’s disease and schizophrenia [41]. GA has been effective against poor blood circulation in the brain and peripheral blood vessels. It is used as a health supplement in the USA and Japan, and as a prescription medication in Germany and France. GA directly binds to activating enzyme E1 and impairs formation of E1-Sumo intermediates [4]. Additionally, in the current work, we have included the Sumoylation agonist Betulinic acid (BA) to authenticate our results. BA has been isolated from *Betula alba*, and is reported to be a Sumoylation agonist. BA inhibits Sp1 expression by enhancing Sp1 Sumoylation [41].

In the present study, we characterized and documented the role and plausible mechanism of small molecule GA, a Sumoylation inhibitor, in rescuing LECs by blocking/abating adverse signaling induced by oxidative stress and mediated by aberrant Sumoylation. 

## 2. Results

### 2.1. GA, a Sumoylation Inhibitor, Augmented the Expression of Sp1 and Prdx6 in LECs

**Figure 1:**
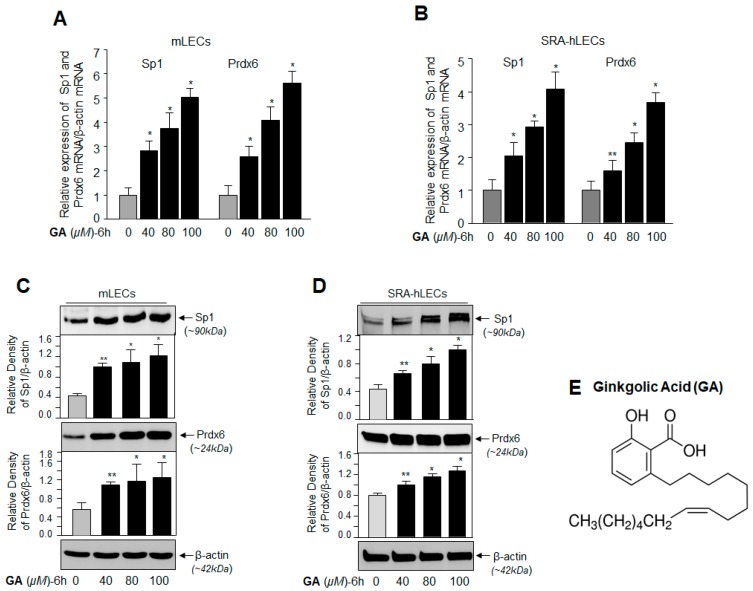
Ginkgolic acid (GA), a Sumoylation inhibitor, enhanced the expression of specificity protein1 (Sp1) and its target gene, Peroxiredoxin 6 (Prdx6), in a dose-dependent manner. (**A**,**B**) Total RNA was extracted from mLECs (**A**) and SRA-hLECs (**B**) treated with different concentrations of GA for 6 hours (h). Equal amounts of RNA were used for cDNA synthesis, and then, quantitative polymerase chain reaction (qPCR) analysis was conducted to measure the levels of Sp1 and Prdx6 using their specific probes. The data represent the mean  ±  SD from three independent experiments. ** *p* < 0.05; * *p* < 0.001. (**C**,**D**) mLECs (**C**) and SRA-hLECs (**D**) were treated with different doses of GA. Total cell lysate was prepared and immunoblotted 6 h later with Sp1 and Prdx6 antibodies, respectively. β-actin was used as loading control. (**E**) Represents GA’s chemical structure.

As Sumoylation dysregulates the abundance of Sp1 and its target gene Prdx6, we sought to determine whether application of GA would modulate the expression levels of Sp1 and its target gene Prdx6 [12]. We assessed expression levels of Sp1 and Prdx6 from their basal expression in mouse LECs (mLECs) treated with GA. After 6 h, we found that GA significantly promoted the expression of Sp1 and Prdx6 mRNA in concentration-dependent fashion (Figure 1A, left panel Sp1 mRNA and right panel Prdx6 mRNA; gray vs. black bars). In another set of GA-treated mLECs (Figure 1C), cellular extract immunoblotted with anti-Sp1 or anti-Prdx6 antibodies revealed a significant increase in expression of both the proteins, with maximum expression levels observed at 80 µM and 100 µM of GA concentration, which was consistent with increased expression of mRNA (Figure 1A). Because expression patterns can differ in cell types of different genetic backgrounds, we next examined whether the results obtained in primary mLECs were reproducible in a human LEC cell line (SRA-hLECs). We found that SRA-hLECs treated with GA (40–100 µM) for 6 h had a pattern of increased expression of transcript or protein of both molecules (Figure 1B,D) similar to that observed in mLECs. These data argue that GA activated expression of Sp1 and Prdx6 by regulating their transcription.

### 2.2. BA, a Sumoylation Agonist, Reduced the Expression of Sp1 and Prdx6 

**Figure 2:**
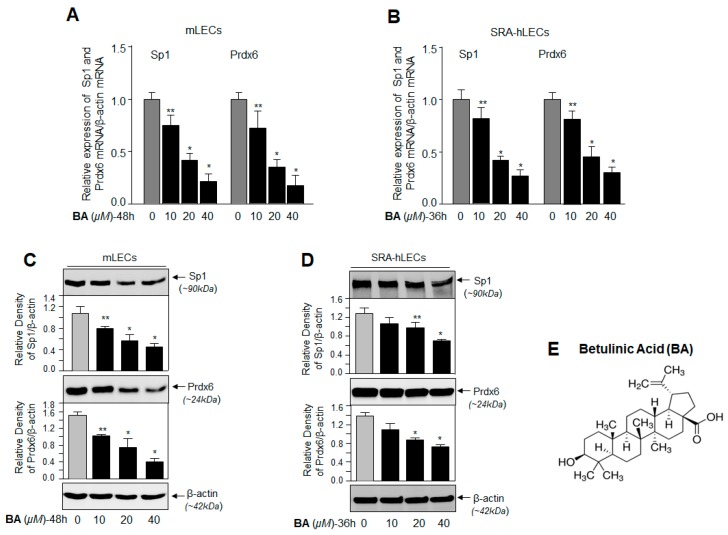
Betulinic acid (BA) reduced Sp1 and Prdx6 expression in a concentration-dependent manner. (**A**,**B**) mLECs (**A**) and SRA-hLECs (**B**) were treated with different doses of BA, and 36–48 h later, total RNA was isolated and processed for cDNA synthesis, followed by an Sp1 and Prdx6 mRNA analysis by Real-Time Reverse Transcriptase-qPCR (RT-qPCR). The data represent mean  ±  SD from three independent experiments. ** *p* < 0.05; * *p* < 0.001. (**C**,**D**) Protein was isolated from mLECs (**C**) and SRA-hLECs (**D**), and treated with different concentrations of BA for 48 h and 36 h, respectively. Equal amounts of protein loaded on SDS-PAGE were immunoblotted with antibodies specific to Sp1 and Prdx6. β-actin served as endogenous control. (**E**) Represents BA’s chemical structure.

A previous study showed that BA decreases Sp1 expression by enhancing Sp1 Sumoylation [42,43]. To examine expression of Sp1 and its target gene Prdx6 at transcription level, we measured mRNA in mLECs and SRA-hLECs treated with different concentrations of BA for 48 h and 36 h, respectively, by quantitative polymerase chain reaction (qPCR). We found a significant reduction in Sp1 and Prdx6 mRNA expression (Figure 2A,B) with BA treatment at concentrations of 20 µM and 40 µM. To check whether the BA affected Sp1 stability in LECs, cellular extracts isolated from LECs treated with BA (10 µM–40 µM) were immunoblotted. While we did not observe a significant difference at a low concentration (10 µM) of BA, concentrations of 20 µM and 40 µM BA significantly reduced the Sp1 and Prdx6 protein levels (Figure 2C,D). The data suggest that GA promoted Sp1 and Prdx6 expression by interfering with Sumoylation signaling.

### 2.3. GA Inhibition of Global Protein Sumoylation Included Sp1 and Prdx6 Sumoylation in LECs In Vivo

**Figure 3:**
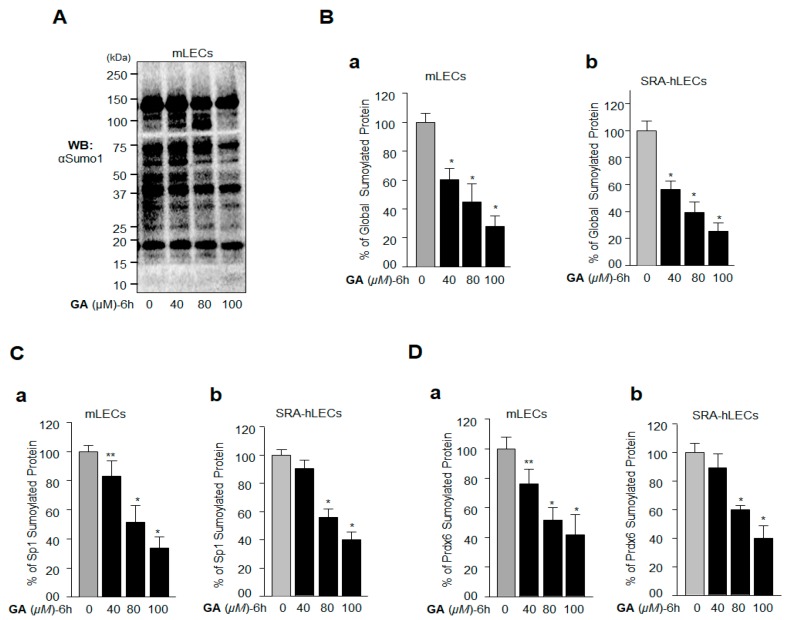
GA treatment inhibited protein Sumoylation in vivo in lens epithelial cells (LECs). (**A**,**B**) GA treatment led to a decrease of total Sumoylated proteins in mLECs and SRA-hLECs. (**A**) Total protein was isolated from mLECs, and treated with different concentrations of GA for 6 h as shown. Samples containing equal amounts of protein content were immunoblotted with the anti-Sumo1 antibody. The protein band below 20 kDa reflected equal loading of protein samples. (**B**) mLECs (a) and SRA-hLECs (b) were treated with different concentrations of GA as shown; 6 h later total cell extract was prepared. Equal amounts of protein were used for the Sumo1 ELISA assay to measure the total protein Sumoylation. The data represent the mean  ± SD from three independent experiments. * *p* < 0.001. (**C**) (a) (mLECs) and (b) (SRA-hLECs) (**D**) (a) (mLECs) and (b) (SRA-hLECs)) GA treatment also inhibited Sumoylation of Sp1 and Prdx6. mLECs and SRA-hLECs were treated with GA as indicated. Lysate was prepared and used 6 h later, for the Sumo1 ELISA assay to measure the levels of protein Sumoylation. Anti-Sp1 and anti-Prdx6 antibodies were used to measure Sumoylation specific to Sp1 (**C**) and Prdx6 (**D**), respectively. The data represent the mean  ±  SD from three independent experiments. ** *p* < 0.05; * *p* < 0.001.

To examine GA’s effect on the modulation of global protein Sumoylation, specifically of Sp1 and Prdx6 in LECs, we immunoblotted cellular extracts from LECs treated with GA (Figure 3A) with the anti-Sumo1 antibody. We observed that the GA treatment efficiently inhibited global Sumoylation at concentrations of 80 µM and 100 µM. Additionally, to authenticate the data, we carried out sensitive Sumoylation enzyme-linked immunosorbent assay (ELISA) with cellular extracts from GA-treated mLECs and SRA-hLECs, as indicated in Figure 3B and described in Materials and Methods. Data analysis revealed significant inhibition of global protein Sumoylation with GA in LECs in vivo. Furthermore, GA treatment enhanced Sp1 and Prdx6 expression levels (Figure 1). Next, we examined whether GA would affect the Sumoylation status of Sp1 and Prdx6 in vivo. We performed in vivo Sumoylation ELISA using anti Sp1 and anti-Prdx6 antibodies, as described in Materials and Methods. We found that GA inhibited Sp1 and Prdx6 Sumoylation in GA-treated mLECs, as well as in SRA-hLECs (Figure 3C,D), demonstrating the ability of GA to inhibit Sp1 and Prdx6 Sumoylation. On the basis of this result, we hypothesized that, since Sp1 is a transregulator of Prdx6, GA may enhance Prdx6 mRNA expression by stabilizing Sp1’s cellular abundance and activity.

### 2.4. BA Amplified Global Protein Sumoylation, Including Endogenous Sp1 and Prdx6 In Vivo 

**Figure 4:**
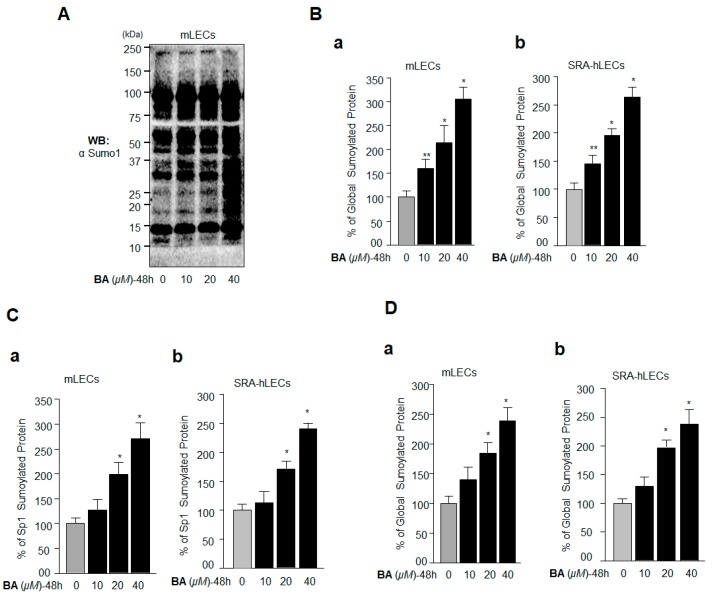
BA amplified global protein Sumoylation including Sp1 and Prdx6 in vivo. (**A**) Aberrant protein Sumoylation by BA in vivo. mLECs were treated with different concentrations of Sumoylation agonist BA for 48 h. Total protein isolated was immunoblotted with anti-Sumo1 antibody and is shown as representative of the result. The protein band of ~15 kDa reflected equal loading of protein samples (**A**). (**B**–**D**) Sensitive Sumo1 ELISA assay showing BA-induced progressive increase in a global manner, as well as Sp1 and Prdx6 Sumoylation in a dose-dependent manner. Protein was isolated from mLECs and SRA-hLECs treated with different concentrations of BA for 48 h, and an equal amount of protein was loaded onto ELISA plate wells coated with anti-Sumo1 antibody. Global protein Sumoylation was quantified (**B**) (a) (mLECs) and (b) (SRA-hLECs)). Sp1 (**C**) (a) (mLECs) and (b) (SRA-hLECs)) and Prdx6 (**D**) (a) (mLECs) and (b) (SRA-hLECs)) Sumoylation levels were measured using anti-Sp1 and anti-Prdx6 antibodies, respectively. The data represent the mean  ±  SD from three independent experiments. ** p < 0.05; * p < 0.001.

Based on the above data (Figure 2) demonstrating that BA reduced the expression of Sp1 and its target gene Prdx6, we postulated that the suppression of Sp1 expression may have been linked to its hyperSumoylation-driven dysregulation, which in turn led to the reduction in Prdx6 mRNA expression. BA has been reported to enhance protein Sumoylation [43], and Sumoylation of Sp1 is known to reduce its cellular abundance due to protein degradation [20], and its mRNA, possibly due to autoregulation [44,45]. As expected, immunoblot analysis of extracts isolated from BA-treated LECs with Sumo1 antibody revealed that BA enhanced Sumo-protein conjugates globally (Figure 4A). We further verified this outcome by applying a sensitive Sumo1 ELISA assay to quantify the status of Sumo1-protein conjugates in BA-treated LECs, as described in Materials and Methods. We observed that cellular extract having equal amounts of protein isolated from mLECs and SRA-hLECs treated with different concentrations of BA showed a significant increase in Sumo1 conjugates (Figure 4B). We also found that the Sumoylation status of Sp1 (Figure 4C) and Prdx6 (Figure 4D) were dramatically enhanced in vivo in these cells, suggesting plausible involvement of hyperSumoylation-mediated dysregulation of Sp1 and Prdx6.

### 2.5. Oxidative Stress-Induced Aberrant Global Sumoylation of Proteins, Including Sp1 and Prdx6, Was Inhibited by GA in LECs In Vivo

**Figure 5:**
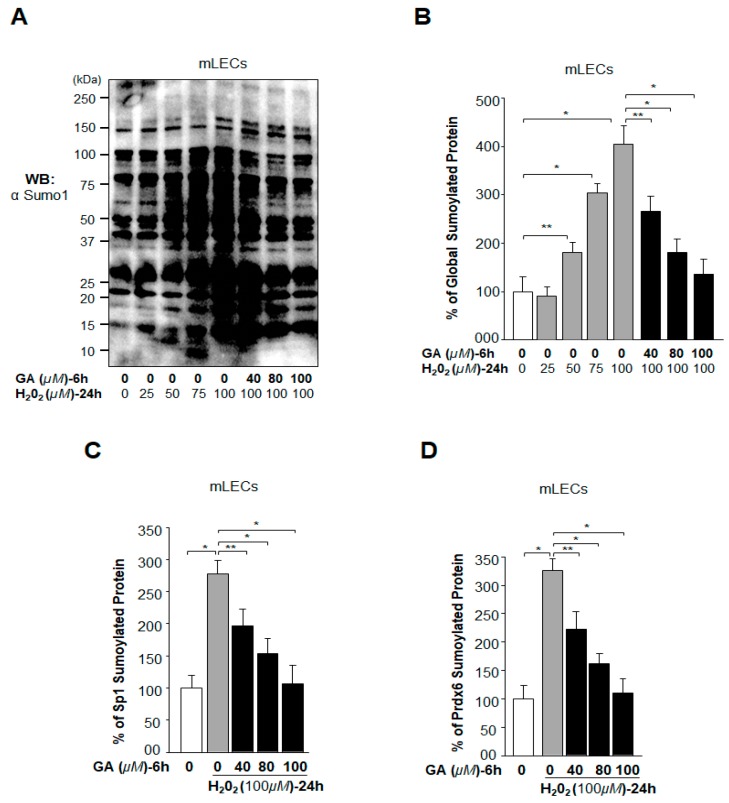
GA application reversed oxidative stress-induced aberrant Sumoylation of proteins in vivo. (**A**,**B**) mLECs were treated with DMSO control or different concentrations of GA for 6 h, and then cells were washed and exposed to H_2_O_2_ as indicated. Total protein was isolated from mLECs treated with GA and/or H_2_O_2_. Samples that had equal amounts of protein content were immunoblotted (**A**) with an anti-Sumo1 antibody. The same protein lysate was used for a Sumo1 ELISA assay to quantitate the level of global protein Sumoylation using an anti-Sumo1 antibody as shown. (**C**,**D**) GA treatment reversed the aberrant Sumoylation of Sp1 and Prdx6 induced by oxidative stress. mLECs pretreated with DMSO or GA were exposed to H_2_O_2_ as indicated. Cell lysate with an equal amount of protein was used for Sumo1-ELISA assay to quantify the level of Sp1 (**C**) and Prdx6 (**D**) protein Sumoylation, using anti-Sp1 or anti-Prdx6 antibodies, respectively. The data represent the mean  ±  SD from three independent experiments. Control (open bar) vs. H_2_O_2_ (gray bar) treated and pretreated with DMSO vs. GA (black bars) in the presence of H_2_O_2_. ** *p* < 0.05; * *p* < 0.001.

Oxidative stress-induced aberrant signaling is known to enhance Sumo conjugation, resulting in cellular abnormalities [13,46]. In the current study, our primary goal was to evaluate whether GA treatment would reverse the oxidative stress-induced aberrant Sumoylation-mediated deleterious signaling. LECs treated or untreated with different concentrations of GA were exposed to H_2_O_2_ as indicated in Figure 5. Cellular extracts were immunoblotted (Figure 5A) and subjected to an in vivo Sumoylation ELISA assay (Figure 5B). Data analysis revealed that a lower concentration (25 μM) of H_2_O_2_ did not significantly influence Sumo1 conjugation, however, higher concentrations (50 µM upwards) did induce Sumo1 conjugation (Figure 5A,B). Noticeably, GA pretreatment significantly blunted the H_2_O_2_-induced aberrant Sumoylation (Figure 5A,B). Because aberrant Sumoylation signaling dysregulates Sp1 and its target gene Prdx6, leading to cellular damage, we next tested whether GA treatment attenuates aberrant Sp1 and Prdx6 Sumoylation in LECs. To analyze the specific Sumoylation status of Sp1 and Prdx6, LECs pretreated with dimethyl sulfoxide (DMSO) and/or GA were exposed to 100 µM of H_2_O_2_ (the most effective concentration observed in Figure 5A). Equal amounts of protein were loaded onto ELISA plate well precoated with anti-Sp1 or anti-Prdx6 antibodies to measure Sp1 Sumoylation or Prdx6 Sumoylation, respectively. Our results revealed that GA did restore the H_2_O_2_ induced aberrant protein Sumoylation (Figure 5A,B), including Sumoylation of Sp1 (Figure 5C) and Prdx6 (Figure 5D). 

### 2.6. GA Attenuated the Erratic Sumoylation Process in SRA-hLECs Overexpressing Sumo1 In Vivo 

**Figure 6:**
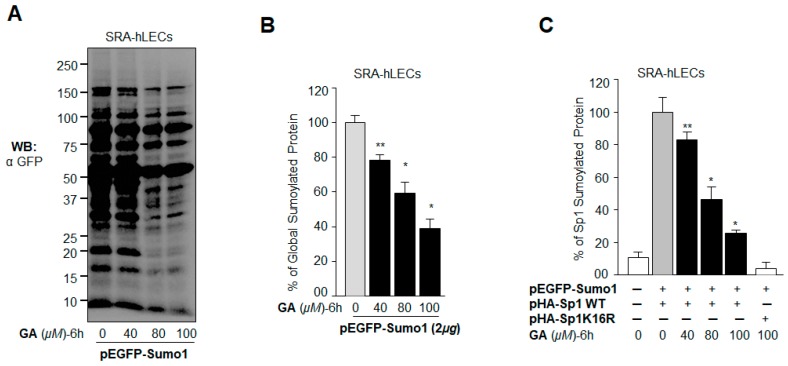
GA blocked aberrant Sumoylation signaling in SRA-hLECs. (**A**) LECs were transfected with pEGFP-Sumo1 and then treated with various concentrations of GA (40–100 µM) for 6 h. Equal amounts of extracted protein containing *N*-ethylmaleimide (NEM) and MG132 were immunoblotted with an anti-GFP antibody. (**B**) The same lysate (as above) was used to quantify global Sumoylation protein. Equal amounts of protein were loaded onto the GFP antibody-coated ELISA plate-well. Following incubation with a Sumo1 antibody, the Sumoylated protein was quantified after the addition of color developing reagent as described in Materials and Methods. (**C**) Inhibition of Sp1 Sumoylation by GA in vivo. LECs transfected with the indicated combinations of pEGFP-Sumo1, Sp1 or a Sumoylation-deficient mutant (Sp1K16R) were treated with various concentrations of GA (40–100 µM) for 6 h. Equal amounts of cell extract were used to quantify exogenous Sp1 Sumoylation using an anti-human Influenza virus hemagglutinin (HA) antibody. The data represent mean ± SD from three independent experiments (** *p* < 0.05; * *p* < 0.001).

A previous experiment (Figure 5) demonstrated that GA did inhibit the protein Sumoylation caused by oxidative stress. However, oxidative stress can have multifaceted effects on cell signaling and Sumoylation. To determine precisely whether GA blunted aberrant Sumoylation of proteins directly in cells overexpressing Sumo (free Sumo1) in vivo, we analyzed GA’s effect on levels of Sumo conjugates, including Sp1, in LECs overexpressing enhanced green fluorescent protein (EGFP) tagged Sumo1. To this end, SRA-hLECs overexpressing EGFP-Sumo1 were treated with different concentrations of GA. Western blot analysis with an anti-GFP antibody showed that GA reduced the levels of Sumo1 conjugation in dose-dependent manner (Figure 6A). Similar results were derived from the sensitive Sumo1 ELISA assay (Figure 6B), demonstrating GA’s potential in blunting aberrant Sumo conjugation globally. We next examined the specific effect of GA on Sp1 Sumoylation, as Sp1 can be modified by Sumo1 in vivo at lysine residue position 16 [20]. We found that the Sp1 Sumoylation level was markedly reduced by GA treatment (Figure 6C), suggesting that GA can restore Sp1’s aberrant Sumoylation-mediated dysregulation. 

### 2.7. BA Further Promoted Protein Sumoylation in SRA-hLECs Overexpressing Sumo1 In Vivo

**Figure 7:**
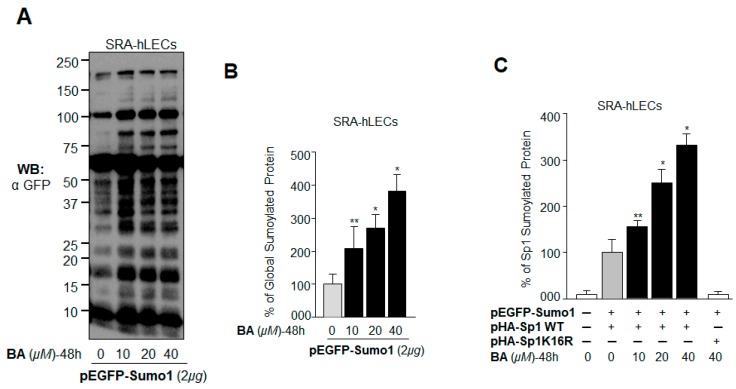
BA promoted aberrant protein Sumoylation in vivo. (**A**) pEGFP-Sumo1 (2 µg) transfected LECs were treated with different concentrations of BA (10–40 µM) for 48 h. Cells were lysed in radio immunoprecipitation assay (RIPA) buffer containing NEM and MG132, and the lysates were separated by sodium dodecyl sulfate polyacrylamide gel electrophoresis (SDS-PAGE), followed by immunoblotting with an anti-GFP antibody. (**B**) The same lysate was used to quantify global Sumoylation protein by Sumo1 ELISA assay. Equal amounts of protein were loaded onto the GFP antibody-coated wells and incubated with the Sumo1 antibody to quantify the level of Sumoylated protein. (**C**) Increased Sp1 Sumoylation by BA in vivo. LECs cotransfected with either pEGFP-Sumo1 plus Sp1 or pEGFP-Sumo1 plus Sp1K16R were treated with different concentrations of BA for 48 h as indicated. A sensitive Sumo1-ELISA assay was used to quantify exogenous Sp1 Sumoylation levels using anti-hemagglutinin (HA) as described in Materials and Methods. The data represent mean ± SD from three independent experiments (** *p* < 0.05; * *p* < 0.001).

We have shown that Sumo1 overexpression reduces expression of the antioxidant Prdx6 and Sp1, and these cells become more susceptible to oxidative damage or apoptotic stimuli [13,14,17]. To examine the effects of BA on Sumoylation of proteins in cells overexpressing Sumo1, LECs were transfected with pEGFP-Sumo1 and then treated with Sumoylation agonist BA. As indicated in Figure 7A, immunoblotting with an anti-GFP antibody demonstrated significantly higher protein Sumoylation, even at a low concentration (10 µM) of BA. We further validated the aberrant Sumoylation signaling by using a sensitive Sumoylation ELISA assay as shown in Figure 7B. To determine specifically whether BA treatment affected the Sumoylation status of Sp1, LECs cotransfected with HA-tagged Sp1 WT and GFP-tagged Sumo1 were treated with different doses of BA and processed by Sumoylation ELISA. As shown in Figure 7C, BA dramatically enhanced Sp1 Sumoylation in dose-dependent fashion, while no change was detectable in the Sumoylation status of Sp1K16R mutated at its Sumoylation site. Taken together, the results showed that BA amplified global protein Sumoylation, including that of Sp1, suggesting that aberrant Sumoylation may play a major role in the reduction of Sp1 expression in LECs (Figure 2), as reported in other cell types [14,41].

### 2.8. Treatment with Sumoylation Inhibitor GA Enhanced Sp1–DNA Binding, while Sumoylation Agonist BA Reduced the Binding

**Figure 8:**
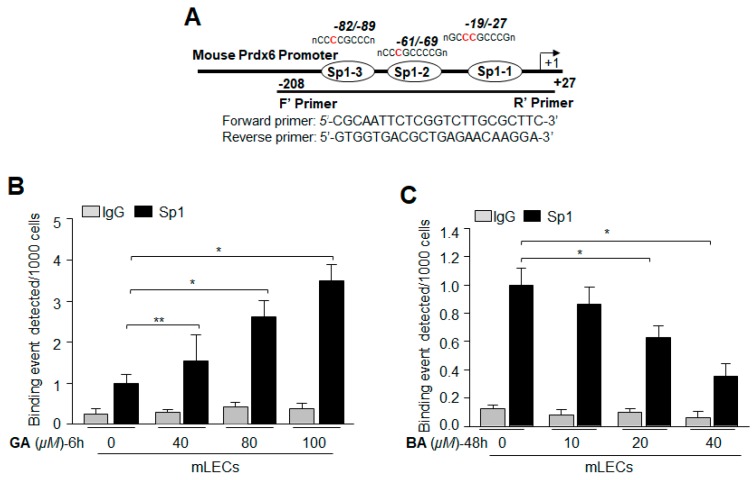
Treatment with Sumoylation inhibitor GA enhanced Sp1–DNA binding, while Sumoylation agonist BA reduced binding activity. (**A**) Digramatic illustration of 5′-proximal promoter region of Prdx6 containing Sp1 binding sites showing primer location and sequences used in ChIP assay. LECs were treated with three different concentrations of GA (**B**) for 6 h or BA (**C**) for 48 h as shown. Chromatin immunoprecipitation assay was carried out with an anti-Sp1 antibody (black bars) and control IgG (gray bars). Pulled DNA fragments were subjected to a qPCR analysis for Sp1 binding site(s) of Prdx6 promoter. GA significantly enhanced the Sp1-DNA enrichment in concentration-dependent fashion. In contrast, BA reduced the Sp1-DNA availability to Sp1 response elements present in Prdx6 promoter. The data represent the mean ± SD from three independent experiments (** *p* < 0.05; * *p* < 0.001).

Given the apparent inductive response of antioxidant Prdx6 and its transregulator Sp1 to GA, we sought to determine the effect of GA at molecular levels: Sp1-DNA interaction in LECs treated with GA. To determine if increased DNA binding activity of Sp1 was due to its increased enrichment by GA in vivo, we carried out chromatin immunoprecipitation (ChIP) assay to measure the occupancy of Sp1 on Prdx6 gene promoter. mLECs treated with GA (40–100 µM) for 6 h were processed for ChIP assay as described in Materials and Methods. Figure 8A shows that Sp1 occupied the Prdx6 promoter containing the GC box (Sp1 binding elements) sequences, and the increased enrichment of Sp1-DNA to the sequence was GA concentration-dependent (Figure 8A). No enrichment was detected with control IgG, pointing to the specificity of Sp1 antibody used in the experiment. The ChIP assays directly revealed GA-induced enrichment of Sp1 to its response element(s) (GC-boxes) present in Prdx6 promoter (Upper panel, diagrammatic illustration). In contrast, BA treatment led to a significant decline in Sp1-DNA binding activity (Figure 8B) in the Prdx6 gene promoter. BA-driven reduction of Sp1 binding to DNA was directly related to BA’s concentration, suggesting that BA-mediated reduced expression of Sp1 (Figure 2) was due to dysregulation of Sp1 induced by BA and mediated by Sumoylation. Taken together, data demonstrate that GA enhanced Sp1 enrichment at GC box sequences by inhibiting Sp1 Sumoylation, and thereby, stabilizing its cellular integrity, while BA reduced the availability of Sp1 to DNA by destabilizing through aberrant Sumoylation. Thus, both GA and BA regulated Prdx6 expression through Sumoylation and deSumoylation of Sp1, as observed in Figure 1 and Figure 2.

### 2.9. GA Significantly Promoted Sp1 Binding to Its Element by Blunting Oxidative Stress-Induced Aberrant Sumoylation Signaling

**Figure 9:**
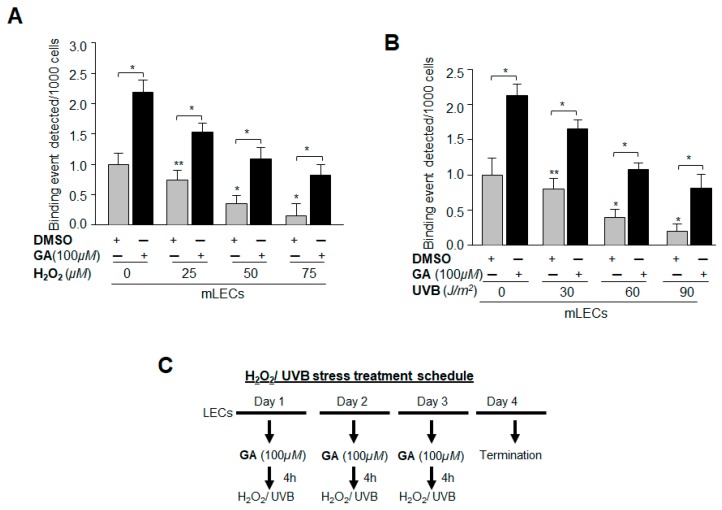
GA significantly promoted Sp1 binding to its element present in Prdx6 promoter by abating oxidative stress and aberrant Sumoylation signaling. (**A**,**B**) mLECs were treated with Sumoylation inhibitor GA. The cells were washed and exposed 4 h later to different concentrations of H_2_O_2_ (**A**) or UVB (**B**) as indicated. Similarly, treatment was repeated on day two and day three. A ChIP assay was carried out 72 h later, using a ChIP grade anti-Sp1 antibody. The DNA fragments were used as templates for qPCR by using a primer designed to amplify −208 to +27 region of the Prdx6 promoter bearing Sp1 sites. Histogram shows the amplified DNA fragment; gray vs. black bars and untreated samples vs. samples treated with H_2_O_2_/UVB. The data represent mean ± SD from three independent experiments (** *p* < 0.05; * *p* < 0.001). (**C**) Represents the treatment schedule.

Data derived from the above experiments convinced us that the increase in Sp1-DNA interaction with GA treatment to its target Prdx6 gene promoter was due to the blunting of aberrant Sumoylation-mediated dysregulation of Sp1. Oxidative stress-induced aberrant Sumoylation-mediated signaling is known to cause disruption of genes/proteins responsible for maintaining cellular homeostasis and cytoprotection [13,14,17]. Thus, we next sought to determine whether GA would restore Sp1-DNA binding activity lost to adverse signaling induced by oxidative stress caused by H_2_O_2_ and/or ultra violet (UV) B, as reported by our group and others [5,6,18,44,47,48]. mLECs were pretreated with DMSO as sham or GA for 4 h, and then exposed to different concentrations of H_2_O_2_ and/or UVB radiation (Figure 9C). ChIP assay was performed as described in Materials and Methods, using Sp1 antibody [12,15,29]. Protein-DNA complex was pulled down from chromatin samples, and immunoprecipitated complex was processed for qPCR using the primers that encompass Prdx6 promoter consisting of Sp1-responsive elements (Figure 8A). A significant decrease was found in the interaction of Sp1 with its target elements at Prdx6 promoter in samples treated with H_2_O_2_ (Figure 9A, gray bars) and/or UVB (Figure 9B, gray bars). GA pretreatment significantly restored the Sp1-DNA interaction (Figure 9A,B, black bars), indicating the ability of GA in correcting oxidative stress-induced Sumoylation-mediated destabilization of Sp1’s DNA binding activity. Data derived from this experiment argue that Sp1 protein-DNA interaction, which is jeopardized in response to oxidative stress and aberrant Sumoylation signaling, can be restored by small molecules such as GA.

### 2.10. Prdx6 Transcription in LECs Was Significantly Potentiated by Sumoylation Inhibitor GA, and Was Inhibited by Sumoylation Agonist BA

**Figure 10:**
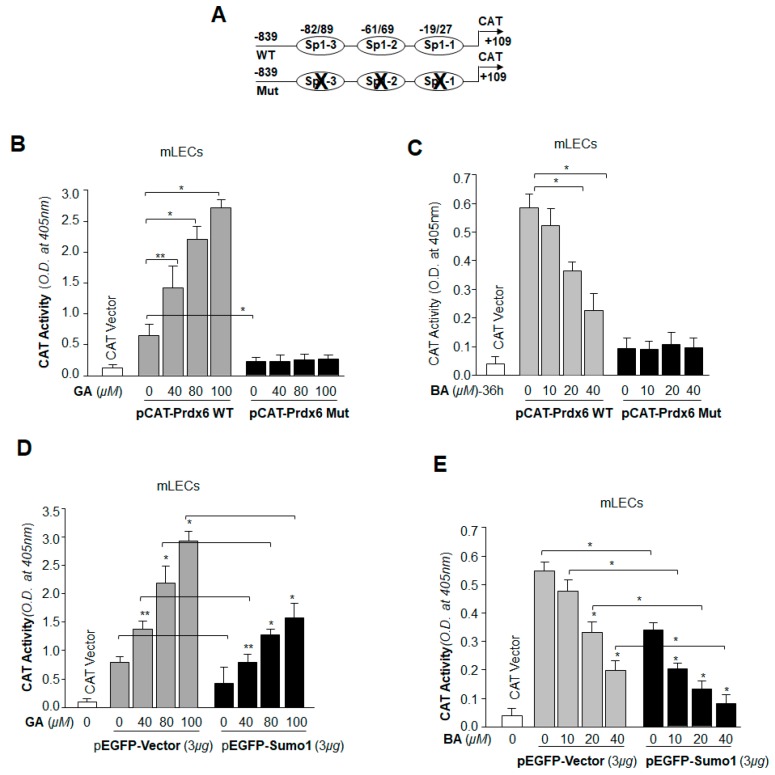
Prdx6 transcription in LECs was significantly increased by Sumoylation inhibitor GA and reduced by Sumoylation agonist BA. (**A**) LECs were transfected with Prdx6 promoter linked to chloramphenicol acetyltransferase (CAT) reporter plasmid (−839/+109) or its mutant (mutated at all Sp1 sites). Transfected cells were treated 48h later, with different concentrations of GA (**B**) for 6 h or BA (**C**) for 36 h as shown. Protein was extracted and CAT-ELISA was performed as stated in Materials and Methods. GA significantly enhanced Prdx6 promoter activity (**B**, gray bars); conversely, BA reduced promoter activity (**C**, gray bars) in dose-dependent manner. The data represent the mean ± SD from three independent experiments (** *p* < 0.05; * *p* < 0.001). (**D**,**E**) Effects of GA and BA in modulation of Prdx6 transcription in cells overexpressing Sumo1. LECs were transfected with Prdx6 promoter linked to the CAT vector (−839/+109) along with either the pEGFP vector or pEGFP-Sumo1 plasmid as indicated. Cells were treated 48 h later with different concentrations of GA (**D**) for 6 h or BA (**E**) for 36 h. Protein was extracted and CAT-ELISA was performed. In the presence of Sumo1, overall promoter activity was reduced, however, activity was restored by GA (**D**, black bars) in comparison to empty vector (**D**, gray bars). Conversely, BA treatment reduced overall promoter activity in dose-dependent manner (**E**, gray bars), and the promoter activity was further decreased in the presence of Sumo1 (**E**, black bars). The data represent the mean ± SD from three independent experiments (** *p* < 0.05; * *p* < 0.001).

We next sought to determine whether the Sp1-DNA binding phenomenon that occurred following GA or BA treatment was functional and would modulate Prdx6 transcription through Sp1. To examine the consequences of the GA- and BA-induced changes in Sp1 binding to its elements on Prdx6 transcription, we transfected mLECs with a pCAT-Prdx6 WT promoter containing all Sp1 sites or its mutant (mutated at all Sp1 binding site[s]) along with a GFP plasmid (Figure 10A). These transfectants were treated with GA (DMSO or 40–100 µM) for 6h and BA (DMSO or 10–40 µM) for 36 h. Transactivation assay with mutant construct showed significant inhibition of chloramphenicol acetyltransferase (CAT) activity, and GA failed to activate it (Figure 10B, black bars). In contrast, wild-type promoter displayed robust promotion of CAT activity in response to GA (Figure 10B, gray bars), suggesting that GA upregulated Prdx6 transcription through GC box elements/Sp1 binding sites. In contrast, BA significantly repressed the WT-Prdx6 promoter activity (Figure 10C, gray bars), and had no significant effect on mutant construct (Figure 10C, black bars), suggesting that BA repressed Prdx6 transcription, a response directly related to Sp1-DNA interaction (Figure 8). 

### 2.11. GA Was Capable of Restoring Prdx6 Transcription Even in Cells Overexpressing Sumo1, while BA Further Reduced the Repression of Prdx6 Transcription 

Next, we examined the ability of GA in restoring Sp1 activity in cells overexpressing Sumo1. This experiment was intended to provide evidence related to the potential use of GA to reduce the effects of Sumoylation-mediated aberrant signaling responsible for pathobiology of cells and tissues [45,47,49,50,51,52,53]. mLECs were transfected with WT-Prdx6 promoter along with GFP-Vector or GFP-Sumo1 plasmid, and then treated with different concentrations of GA (Figure 10D) and BA (Figure 10E). Sumo1 overexpression significantly reduced Prdx6 promoter activity. As expected, GA treatment enhanced the promoter activity in LECs overexpressing Sumo1. We noticed that LECs overexpressing GFP-Sumo1 (Figure 10D, black bars) showed low promoter activity in comparison to pEGFP-Vector (Figure 10D, gray bars). However, promoter activity was significantly high in the presence of GA in those LECs. BA repressed Prdx6 promoter activity (Figure 10E), and the activity was further reduced in LECs overexpressing Sumo1 following BA treatment (Figure 10E). Taken together, our data revealed that GA enhanced Prdx6 transcription even in the presence of excess Sumo1, while BA reduced Prdx6 promoter activity with an abundance of free/excess Sumo1. Findings suggest that GA may be an ideal therapeutic small molecule to combat aberrant Sumoylation-mediated dysregulation of genes/proteins essential for cellular homeostasis.

### 2.12. GA Delivery Blunted Oxidative Stress-Induced Deleterious Signaling-Mediated Injuries to LECs

**Figure 11:**
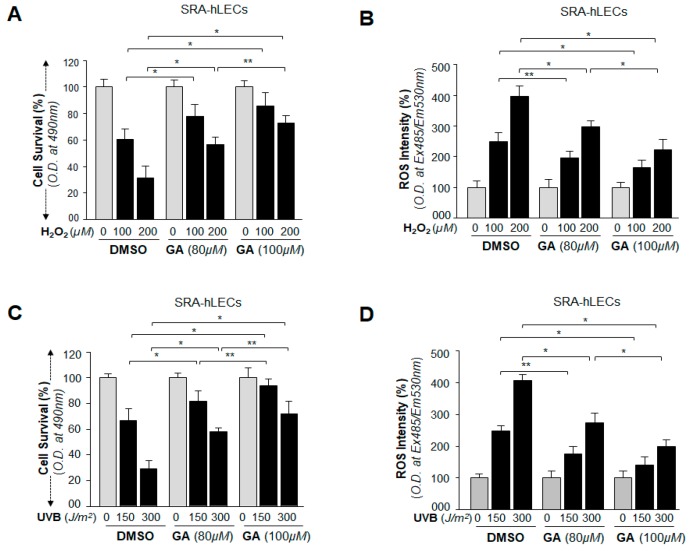
GA application protected LECs by blunting oxidative stress-mediated adverse signaling. (**A**,**B**) SRA-hLECs were pretreated with GA. Cells were washed and replaced 6 h later in Dulbecco’s modified Eagle’s medium (DMEM) (serum free) media containing the different concentrations of H_2_O_2_ as indicated. 3-(4,5-dimethylthiazol-2-yl)-5-(3-carboxymethoxyphenyl)-2 to 4-sulphophenyl) 2H-tetrazolium salt; (MTS) (18 h) and ROS (8 h) intensity were measured. Data are the mean ± SD of three independent experiments. ** *p* < 0.05; * *p* < 0.001 versus control. (**C**,**D**) SRA-hLECs were pretreated with different concentrations of GA for 6 h; cells were washed and exposed to different doses of UVB as indicated. MTS assay (**C**) and ROS intensity (**D**) were quantified after 18 h and 8 h of UVB exposure, respectively. Data are the mean ± SD of three independent experiments. ** *p* < 0.05; * *p* < 0.001 versus control.

Our primary goal was to characterize small molecules that can block oxidative stress and aberrant Sumoylation signaling by enhancing the expression and activity of endogenous Sp1 transcriptional protein and its target protective gene Prdx6. We chose GA because it is known to inhibit aberrant Sumoylation signaling during aging and oxidative stress and to prevent many diseases, such as neurological disease, cardiovascular disease, cancer, macular degeneration, etc. [4,41,54,55,56,57,58,59]. Cells facing oxidative stress induced by environmental stressors generate H_2_O_2_ endogenously, and eyes are maximally exposed to UVB radiation. Therefore, to provide a proof of concept, we examined the efficacy of GA in remedying the cellular injuries evoked by H_2_O_2_ and UVB stress. We found that SRA-hLECs pretreated with GA, as indicated in Figure 11, showed enhanced viability (Figure 11A) of SRA-hLECs and reduced expression of ROS (Figure 11B) during oxidative stress induced by H_2_O_2_ exposure (100 µM and 200 µM). Similarly, significantly increased viability (Figure 11C) and reduced ROS production (Figure 11D) were observed in SRA-hLECs pretreated with GA (80 µM and 100 µM) in oxidative stress induced via UVB radiation (0, 150 and 300 J/m^2^). The results indicated that GA may be considered as a therapeutic small molecule to combat or delay oxidative-induced deleterious signaling and its associated pathobiology caused by stressors.

## 3. Discussion

Changes in cellular microenvironment in response to external or internal cues stimulate cellular transduction that regulates posttranslational modifications, such as Sumoylation, phosphorylation, acetylation, ubiquitination and so on. Sumoylation of transcription factors has been shown to modulate their activity and that modification dynamically alters their target gene expression. Sp1 is a ubiquitous transcription factor which also acts as transactivator of several genes [12,13,14,29,40,60]. This nuclear protein can be a target for various modifications, including Sumoylation [13,14,20,53,61,62]. Several studies have shown that Sumo conjugation to transcription factors or chromatin regulators has negative effects on their integrity and activities [13,14,17,63,64,65,66,67,68]. Increased accumulation of ROS and their damaging effects are linked to the etiopathology and progression of many diseases, such as age-related disorders, neurodegenerative disorders, diabetes, cancer, macular degeneration, cataractogenesis and so on [38,69,70,71,72]. In previous studies we found that oxidative stress-induced aberrant Sumoylation is a major adverse process which affects the status of protein Sumoylation, and is involved in dysregulation of protective molecules, such as Prdx6, transcription factor lens epithelial derived growth factor (LEDGF) and Sp1 [13,14,17,40]. Sumoylation is a reversible dynamic process. We planned to take advantage of this reversibility to test the potential of small molecules such as GA to counteract the aberrant Sumoylation signaling. We observed that treatment of LECs with GA significantly enhanced the expression of Sp1 and its target gene Prdx6. We found that the protein and mRNA expression levels of Sp1 and Prdx6 were dramatically increased in both mLECs and SRA-hLECs treated with GA (Figure 1); conversely, treatment with the Sumoylation agonist BA [43] significantly reduced the expression levels of both molecules (Figure 2), and inhibited LEC growth (data not shown). GA has been reported to have anti-bacterial [73], anti-HIV [74], anti-cancer [44] and neuroprotective activity [41]. GA exerts its effects by deSumoylating proteins, and it does so by blocking the Sumoylation process. The Sumo target is a lysine residue that occurs in the consensus motif; ψKXE, where ψ is a hydrophobic amino acid and X can be any amino acid residues. The Sumoylation pathway is analogous to ubiquitination and requires a specific E1-activating enzyme (SAE1/SAE2), Ubc9, a Sumo-specific E2-conjugating enzyme, and ligating enzyme E3. GA directly binds to E1 activating enzyme and inhibits the Sumoylation pathway [4]. We surmised the increase in expression of the above-noted genes might be linked to reduction in Sumoylation status caused by GA treatment, as both Sp1 and Prdx6 are targets for Sumo1 conjugation, and Sumoylation of these proteins leads to their dysregulation [40]. Furthermore, global protein Sumoylation analysis of LECs treated with GA showed a significant inhibition in the global Sumoylation status as shown in Figure 3. It has been reported that cells treated with GA show a dramatic global reduction in Sumo conjugates [48,73]. The major goal of our study was to determine whether GA has the ability to restore Sumoylation-mediated dysregulation of Sp1 and repression of its target gene Prdx6 [13,20,40,62]. We found that GA treatment significantly inhibited the Sumoylation of Sp1 and Prdx6. It has been reported that GA treatment inhibits RanGap1-C2 Sumoylation in vivo and in vitro [4]. GA has also been found to inhibit Sumoylation of NEMO (a modulator of NF-kB) and reduce IkB-α degradation in breast cancer cells [59]. However, we found that BA significantly enhanced global protein Sumoylation, including Sp1 and Prdx6 (Figure 4). This result is consistent with an earlier report showing that BA reduces the Sp1 level to inhibit lung cancer growth by hyperSumoylating Sp1 [43]. 

Recent studies by others, as well by our group, have shown that oxidative stress enhances Sumo conjugation [13,14,46]. Additionally, we have reported that oxidative stress enhances global Sumoylation, including Sp1 and Prdx6 Sumoylation; those cells are highly susceptible to oxidative damage, however, they can be rescued by the application of Sumoylation-deficient Prdx6 or Sp1 [13,14,40]. A prime aim of this study was to assess the ability of GA to reverse oxidative stress-evoked aberrant Sumoylation-mediated adverse signaling and provide a proof of concept that a small molecule such as GA might be considered to block such damaging signaling. As shown in Figure 5, GA pretreatment globally blunted the H_2_O_2_-induced aberrant levels of Sumoylation of proteins in LECs, suggesting that GA compound has the ability to reverse the adverse phenomena evoked by oxidative stress-mediated aberrant Sumoylation signaling, at least in LECs. However, in acute kidney injury (AKI), Sumoylation plays a cytoprotective role, and GA pretreatment suppresses the Sumoylation induced by Cisplatin nephrotoxicity AKI in mice [22]. This discrepancy of outcomes might be related to cell/tissue type and cellular microenvironment. Sumoylation of cyclin-dependent kinases (CDKs) is known to be involved in the regulation of cell cycle progression, and GA has been shown to deSumoylate the CDKs and alter their activity [75]. Aberrant expression of Sumo1 leads to increased ROS accumulation and reduced antioxidant activity due to degradation of the target protein [13,14]. We think that adverse changes in cellular homeostasis that lead to abnormalities in cells are linked to ROS-induced oxidative stress and aberrant Sumoylation signaling. 

Oxidative stress can induce multiple forms of deleterious signaling. Thus, to understand precisely the role of aberrant Sumoylation in the dysregulation of protein activity, we examined the fate of cellular proteins in LECs overexpressing Sumo1 and tested efficacy of GA. As shown in Figure 6, Western analysis with anti-GFP antibody and Sumo1 ELISA assay revealed that, indeed, GA treatment reversed the overall aberrant Sumoylation of proteins in the presence of abundant Sumo1. Furthermore, Sp1 can be modified by Sumo 1 in vivo at lysine (K) 16 residue [20,40]. We found that the level of Sumoylated Sp1 was markedly reduced by GA treatment (Figure 6), and Sumoylation-deficient Sp1K16R showed resistance against Sumoylation. Conversely, BA increased global Sumoylation that aberrantly further increased Sumo substrates in cells overexpressing Sumo1, including Sp1 (Figure 7), supporting the finding that GA is efficacious in blunting the overstimulated Sumoylation process.

Furthermore, our in vivo DNA binding experiments, at physiological levels as well as in cells facing oxidative stressors, revealed an increased binding of Sp1 at its responsive elements present in Prdx6 promoter in GA-treated LECs (Figure 8 and Figure 9), and that binding was directly related to Prdx6 transcription (Figure 10). In contrast, in BA-treated LECs, Sp1 occupancy was dramatically decreased with a decrease of Prdx6 transcriptional activity (Figure 8 and Figure 10), suggesting that a major event was GA-mediated interruption of Sumoylation leading to cellular abundance of deSumoylated Sp1. Recent reports have shown that the Sumoylation of Sp1 destabilizes its cellular steady state, leading to reduced abundance due to Sumo1-mediated degradation [20,40]. Furthermore, eyes are highly exposed to environmental stressors and sunlight (UV radiation), and H_2_O_2_ is a major culprit in oxidative stress-induced damage of the eye lens/LECs. LECs pretreated with GA showed resistance against H_2_O_2_ and UVB injuries (Figure 11). We believe that GA protects against such injuries by activation of the Sp1-DNA pathways. Sp1 is an auto-regulator and transregulator of Prdx6. Our current study showed that GA application in cells stabilized the cellular state of Sp1, which had been dysregulated due to aberrant Sumoylation and in turn auto-regulated its own transcription and cellular abundance leading to increased Prdx6 expression and cytoprotection.

## 4. Materials and Methods 

### 4.1. Cell Culture 

SRA-hLECs were derived from 12 infants who underwent surgery for retinopathy of prematurity [60] (a kind gift of Dr. Venkat N. Reddy, Eye Research Institute, Oakland University, Rochester, MI, USA). The human LECs cell line (SRA01/04) has been immortalized with SV40. These cells were maintained in DMEM with 15% fetal bovine serum (FBS), 100 µg/mL streptomycin, and 100 µg/mL penicillin in 5% CO_2_ environment at 37 °C as described previously [13,76]. Primary cultured LECs were generated from 6 weekd-old Balb/C mice (*n* = 8) and maintained in DMEM with 10% FBS, as described earlier [77]. To examine the effect of GA (C15:1), cells were treated with different concentrations (40, 80 or 100 µM in complete medium) of GA. A stock solution of GA (20 mM) was prepared in DMSO and diluted in culture medium keeping the final DMSO concentration at <0.05%, and the same concentration of DMSO was used as vehicle control. GA (catalog no. 75741) and BA (catalog no. 855057) were purchased from Sigma Aldrich (St. Louis, MO, USA).

### 4.2. Total Cell Extraction

Total cell extract was prepared. Briefly, LECs (1 × 10^6^) were cultured in 100-mm plates. The cells were washed gently with chilled phosphate-buffered saline (pH 7.4). Cells were collected by centrifugation using a micro-centrifuge and resuspended in three pellet volumes of RIPA buffer [(1% (*v/v*) Nonidet P-40, 0.5% sodium deoxycholate, 0.1% SDS, 0.5 mM phenylmethylsulfonyl fluoride, and protease inhibitor cocktail]. Cells were gently homogenized and made monolayer by passing through syringe following incubation on ice for 30 minutes (min) and centrifugation (4 °C) at 10,000 rpm for 15 min. The supernatant was carefully transferred into fresh tubes from the pellet and individual aliquots were stored at −70 °C to avoid repeated freezing and thawing of the preparation. Protein was estimated according to the Bradford protein assay and/or Pierce^TM^ BCA Protein assay (Thermo Fisher Scientific, Waltham, MA, USA). 

### 4.3. Protein Expression Analysis

Cell lysates of SRA-hLECs and mLECs were prepared in an ice-cold radioimmune precipitation buffer, and protein blot analysis was performed as described previously [78,79,80]. The membranes were probed with anti-HA (ab 18181 and ab9110, Abcam^®^, Cambridge, MA, USA), Anti-Sp1, Anti-Prdx6 antibody (LF-PA0011 and LF-MA0018, Ab Frontier, South Korea), or β-actin (A2066, Sigma-Aldrich, St. Loius, MO, USA) as internal control to monitor those protein expressions. After secondary antibody (sc-2354 and sc-2768, Santa Cruz Biotechnology, Dallas, TX, USA), protein bands were visualized by incubating the membrane with luminol reagent (sc-2048; Santa Cruz, Santa Cruz Biotechnology, Dallas, TX, USA) and images were recorded with a FUJIFILM-LAS-4000 luminescent image analyzer (FUJIFILM Medical Systems Inc., Hanover Park, IL, USA). 

### 4.4. RT-qPCR

Total RNA from the cultured SRA-hLECs and mLECs was isolated using the single-step guanidine thiocyanate/phenol/chloroform extraction method (Trizol Reagent, Invitrogen, Carlsbad, CA, USA). To examine the levels of Sp1 and Prdx6, 0.5 to 2 micrograms of total RNA was converted to cDNA using Superscript II RNAase H-reverse-transcriptase. RT-qPCR was performed with the SYBR Green Master Mix (Roche Diagnostic Corporation, Indianapolis, IN, USA) in a Roche^®^ LC480 Sequence detector system (Roche Diagnostic Corporation). PCR conditions of 10 min hot start at 95 °C, followed by 45 cycles of 10 seconds (s) at 95 °C, 30 s at 60 °C and 10 s at 72 °C. The primer Sequence was: Prdx6 (Human), Forward: 5′-GCATCCGTTTCCACGACT-3′ and Reverse: 5′-TGCACACTGGGGTAAAGTCC-3′; Sp1 (Human), Forward: 5′-CCTGGATGAGGCACTTCTGT-3′ and Reverse: 5′-GCCTGGGCTTCAAGGATT-3′; β-actin (Human), Forward: 5′-CCAACCGCGAGAAGATGA-3′ and Reverse: 5′-CCAGAGGCGTACAGGGATAG-3′;Prdx6 (Mouse), Forward: 5′-TTTCAATAGACAGTGTTGAGGATCA-3′ and Reverse: 5′-CGTGGG-TGTTTCACCATTG-3′; Sp1 (Mouse), Forward: 5′-ATGCCCCTATTGCAAAGACA-3′ and Reverse: 5′-TGGATGTGACAAATGTGCTG-3′; β-actin (Mouse), Forward: 5′-CTAAGGCCAACCGTGAAA-AG-3′ and Reverse: 5′-ACCAGAGGCATACAGGGACA-3′. The relative quantity of the mRNA was obtained using the comparative threshold cycle (CT) method. The expression levels of target genes were normalized to the levels of β-actin as an endogenous control in each group. 

### 4.5. Sumoylation ELISA Assay

An assay of global, Sp1 and Prdx6 Sumoylated form was performed by the EpiQuik in vivo universal protein Sumoylation assay kit (Epigentek, Farmingdale, NY, USA) in accordance with the manufacturer’s instructions. Briefly, in the ELISA assay, total cell lysates were prepared from SRA-hLECs and mLECs (transfected with different plasmid constructs as indicated in figures). Equal amounts of proteins from the cell extracts were added to the strip wells, which were precoated overnight with either anti-Sumo1, anti-GFP, anti-Sp1, anti-Prdx6 or anti-HA serum. They were then incubated in blocking buffer for 45 min, washed three times and incubated with Sumo assay buffer for 1 h at room temperature. After three washes, a Sumo1 antibody was added and the proteins were incubated for 15 min at room temperature. Subsequent to color development by a Sumo detection system, A450nm was measured as described in our published protocol [13,14]. 

### 4.6. ChIP Assay

ChIP was performed using the ChIP-IT^®^ Express (Cat. No. 53008; Active Motif, Carlsbad, CA, USA) and ChIP-IT^®^ qPCR analysis kit (Cat. No. 53029; Active Motif, Carlsbad, CA, USA) following the manufacturer’s protocol [29]. The following antibodies were used: control IgG and antibody specific to Sp1 (ab13370, Abcam, Cambridge, MA, USA) and/or HA (ab9110 and ab18181, Abcam). RT-PCR or real-time qPCR amplification was carried out using 5 μL of DNA sample with primers [mouse promoter bearing Sp1 sites, forward primer: 5′-CGCAATTCTCGGTCTTGCGCTTC-3′ and reverse primer: 5′-GTGGTG-ACGCTGAGAACAAGGA-3′, positions −208/+27; and human promoter within Sp1 binding sites, forward primer: 5′-CATCACGTGTGCAGAGACGGC-3′ and reverse primer: 5′-CACGTCCCCGA-GAAGCAGAC-3′, positions −342/+30 relative to the A in the ATG translation initiation codon] specific to the Prdx6 promoter. The program for quantification amplification was 3 min at 94 °C, 20 s at 95 °C, 30 s at 59 °C and 30 s at 72 °C for 36 cycles in 25 μL reaction volume (RT-PCR) or 2 min at 95 °C, 15 s at 95 °C, 20 s at 58 °C and 20 s at 72 °C for 40 cycles in 20 μL reaction volume (qPCR). Data were obtained with RT-PCR run on 1% agarose gel, with band visualized under UV and image captured. Data obtained with qPCR were plotted and presented in the form of a histogram.

### 4.7. Plasmid and Constructs Detail

#### 4.7.1. Construction of pEGFP-Sumo1

For eukaryotic expression, the full length of Sumo1 cDNA was subcloned into a pEGFP-C1 vector. The coding region of Sumo1 was amplified by PCR from human lens cDNA library using a forward (5′-CCGTCGACATGTCTGACCAGGAG-3′) and reverse primer (5′-TCGGATCCGTTTTGAACACC ACA-3′) with restriction enzyme sites, SalI and BamHI. The PCR product was digested and ligated into pEGFP-vector. All the transfection experiments were carried out with either Superfactamine Reagent (Invitrogen, Carlsbad, CA, USA) or the Neon Transfection system (Invitrogen). The HA-tagged Sp1 construct [pClneo-HA-Sp1] was a gift from Dr. Hans Rotheneder (University of Vienna, Austria). pClneo-HA-Sp1K16R was generated by Site-directed mutagenesis [40,81].

#### 4.7.2. Site-Directed Mutagenesis (SDM) 

PCR-based site-directed mutagenesis was carried out using the QuikChangeTM lightning site-directed mutagenesis kit (Agilent Technologies; Catalog No. 210518), following the company’s protocol. Briefly, amino acid exchanges K16R in Sp1 were generated by point mutations in the pCl-neo-HA-Sp1 constructs. The following complementary primers, forward primer: 5′-GCTGTGGTGA**GG**ATTGAAAAAGGAGTTGGTGGC-3′ and reverse primer: 5′-GCCACC AACTCCTTTTTAAAT**CC**TCACCACAGC-3′ were used (changed nucleotides are in boldface type and underlined).

XL10-Gold ultra-competent cells (Agilent Technologies) were transformed with the resultant plasmid. The plasmid was amplified, and the mutation was confirmed by sequencing as described previously [82].

### 4.8. CAT Reporter Assay

#### 4.8.1. Construction of Human Prdx6 Promoter-CAT Reporter Vector

The 5′-flanking region (−839 to +109 bp) was isolated from mouse genomic DNA and sequenced. A construct of −839 bp was prepared by ligating it to basic pCAT vector (Promega) using the SacI and XhoI sites. The plasmid was amplified and used for the CAT assay. Primers used were as follows: 5′-CTTCCTCTGGAGCTCAGAATTTAC-3′ and 5′-CAGGAACTCGAGGAAGCGGAT-3′. Sp1 mutants constructs were generated by point mutations in the Prdx6–CAT plasmid using the QuikChangeTM lightning site-directed mutagenesis kit (Agilent Technologies; Catalog No. 210518) as described previously [12]. 

#### 4.8.2. Cotransfection and Prdx6 Promoter Activity Assay

CAT assay was performed using a CAT-ELISA kit (Roche Diagnostic Corporation, Indianapolis, IN, USA). Cells were transfected with a reporter construct (pCAT-Prdx6), and treated with different concentrations of GA (6 h) and BA (36 h) as indicated in figures. After incubation, cells were harvested, extracts were prepared and protein was normalized. CAT-ELISA was performed to monitor CAT activity in accordance with the manufacturer’s instructions. A_405_ was measured using a microliter plate ELISA reader. Transactivation activities were adjusted for transfection efficiencies using GFP values cotransfected during transfection assays.

### 4.9. Quantitation of Intracellular ROS Level by H2-DCF-DA 

Intracellular ROS level was measured by use of fluorescent dye dichlorofluorescin diacetate (H2DCFDA), a nonpolar compound that is converted into a polar derivative (dichlorofluorescein) by cellular esterase after incorporation into cells [71]. On the day of the experiment, the medium was replaced with Hank’s solution containing 10 µM H2DCFDA dye and cells were incubated. After 30 min, intracellular fluorescence was detected with excitation at 485 nm and emission at 530 nm by a Spectra Max Gemini EM (Mol. Devices, Sunnyvale, CA, USA). 

### 4.10. Cell Survival Assay (MTS Assay)

A colorimetric MTS assay (Promega, Madison, WI, USA) was performed as described earlier [71,83,84]. This assay of cellular viability uses MTS and an electrone coupling reagent (Phenazine ethosulfate; PES). PES has enhanced chemical stability, which allows it to be combined with MTS to form a stable solution. Assays are performed by adding MTS reagent directly to culture cells, incubating for 1–4 h and then recording absorbance at 490 nm with a 96-well plate reader, Spectra Max Gemini EM (Mol. Devices, Sunnyvale, CA, USA). Results were normalized with absorbance of the untreated control(s). 

### 4.11. Induction of Ultraviolet (UV) B and Hydrogen Peroxide (H_2_O_2_) Induced Stress

For UVB treatment, mLECs and SRA-hLECs were pre-cultured for 16 h in 100 mm petri dishes with DMEM-10% and 15% FBS. Cells were pretreated with GA for 4–6 h as indicated in figures. Cells were washed twice and the medium was replaced with phosphate buffered saline (PBS, pH 7.2). The plates containing the monolayers were exposed to UVB using UV lamp emitting 270–320 nm peaking at 302 nm wavelength (UVP, Upland, CA, USA) as indicated in figures. The energy actually incident onto the working area was measured by a UVX Radiometer (UVP Inc., Upland, CA, USA) and expressed in J/m^2^. The UV dosage of J/m^2^ was selected on the basis of results from our previous work [40]. After irradiation, PBS was withdrawn and fresh medium was added. At intervals of 8 h and 18 h later, ROS and MTS assays were performed to monitor the levels of ROS and cell viability, and the percentages of ROS and cell survival levels were calculated for each group. 

For H_2_O_2_ treatment, mLECs and SRA-hLECs were pre-cultured for 16 h in 100 mm petri dishes with DMEM-10% and 15% FBS. Cells were treated with different concentrations of GA; 6 h later cells were washed twice with PBS, and the medium (0.2% BSA + DMEM) was replaced with predefined concentrations of H_2_O_2_. After 24 h total protein was isolated and equal amounts of protein were used for western blot analysis and/or Sumo1 specific ELISA assay to measure the level of Sumoylated forms of proteins. ROS (8 h) and MTS (18 h) assays were performed to monitor the levels of ROS and cell viability, and the percentages of ROS and cell survival levels were calculated for each group. To detect Sp1-DNA binding events, cells cultured in 100 mm plates were pretreated with GA, and 4 h later subjected to H_2_O_2_ (0, 25, 50 and 75 µM) and/or UVB (0, 30, 60, 90 J/m^2^) as indicated in Figure 9C. Similarly, treatment was repeated on day two and day three. After 72 h, ChIP assay was carried out using ChIP grade anti-Sp1 antibody.

### 4.12. Statistical Methods

For all quantitative data collected, statistical analysis was conducted by Student’s *t* test and/or one-way ANOVA when appropriate, and was presented as mean ± SD of the indicated number of experiments. A significant difference between control and treatment groups was defined as *p* value of <0.05 and 0.001 for two or more independent experiments. 

## 5. Conclusions

**Figure 12:**
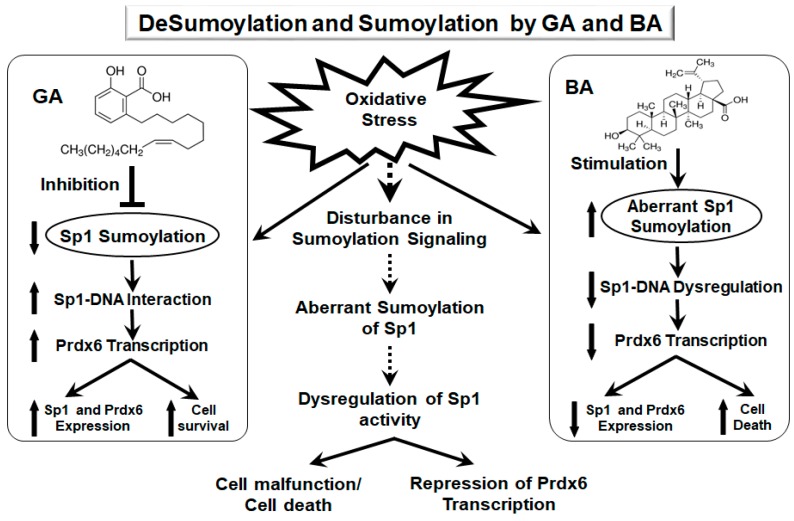
Diagrammatic illustration showing plausible mode of action of GA and BA in modulation of Sp1 and its targeted gene Prdx6 and in determining LECs fate. Oxidative stress-evoked aberrant Sumoylation signaling causing dysregulation of Sp1 activity and Prdx6 in LECs can be blunted by GA application. GA enhances Sp1 transcriptional activity by enhancing its cellular stability through inhibiting its aberrant Sumoylation.

Our work, for the first time, has mechanistically demonstrated that GA, a Sumoylation inhibitor, protects LECS by augmenting the expression and stability of Sp1 and its antioxidant gene Prdx6. GA acts by negatively regulating oxidative stress-induced aberrant Sumoylation signaling that leads to hyperSumoylation-mediated dysregulation of Sp1 expression (mRNA and protein) and activity, thereby resulting in suppression of its target protective protein Prdx6. We also showed that GA treatment restored Sp1’s transcriptional activity by enhancing its enrichment at its response elements present in Prdx6 promoter, thereby promoting Prdx6 transcription. The findings of this study may provide a foundation for a strategy to apply small molecules such as GA to repair or treat adverse signaling evoked by oxidative stress and aberrant Sumoylation (Figure 12).

## Abbreviations

Sp1	Specificity protein 1
Prdx6	Peroxiredoxin 6
GA	Ginkgolic acid
LECs	Lens epithelial cells
BA	Betulinic acid
SUMO	Small ubiquitin-related modifier
ROS	reactive oxygen species
Senps	Sumo-specific proteases
Prdxs	Peroxiredoxins
Cys	Cysteine
GSH	glutathione
PLA_2_	phospholipase A_2_
mLECs	mouse LECs
SRA-hLECs	human LEC cell line
h	hours
qPCR	quantitive polymerase chain reaction
RT-qPCR	real-time qeverse transcriptase-quantitive polymerase chain reaction
ELISA	enzyme-linked immunosorbent assay
DMSO	Dimethyl Sulfoxide
EGFP	enhanced green fluorescent protein
NEM	*N*-ethylmaleimide
HA	hemagglutinin
RIPA	radio immunoprecipitation assay
SDS-PAGE	sodium dodecyl sulfate polyacrylamide gel electrophoresis
ChIP	chromatin immunoprecipitation
UV	Ultra violet
CAT	chloramphenicol acetyltransferase
DMEM	Dulbecco’s modified Eagle’s medium
MTS	3-(4,5-dimethylthiazol-2-yl)-5-(3-carboxymethoxyphenyl)-2 to 4-sulphophenyl) 2H-tetrazolium salt
LEDGF	lens epithelial derived growth factor
AKI	acute kidney injury
CDKs	cyclin-dependent kinases
FBS	fetal bovine serum
min	minutes
s	seconds
DTT	1,4-Dithiothreitol
CT	threshold cycle
OD	optical density
sec	seconds
SDM	Site-directed mutagenesis
H2DCFDA	dichlorofluorescin diacetate
PES	Phenazine ethosulfate
